# Amino acid supplementation and impact on immune function in the context of exercise

**DOI:** 10.1186/s12970-014-0061-8

**Published:** 2014-12-14

**Authors:** Vinicius Fernandes Cruzat, Maurício Krause, Philip Newsholme

**Affiliations:** CHIRI Biosciences Research Precinct, Faculty of Health Sciences, School of Biomedical Sciences, Curtin University, Perth, Western Australia Australia; Laboratory of Cellular Physiology, Department of Physiology, Institute of Basic Health Sciences, Federal University of Rio Grande do Sul, Porto Alegre, RS Brazil

**Keywords:** Immunonutrition, L-glutamine, L-arginine, L-leucine, Oxidative stress

## Abstract

Moderate and chronic bouts of exercise may lead to positive metabolic, molecular, and morphological adaptations, improving health. Although exercise training stimulates the production of reactive oxygen species (ROS), their overall intracellular concentration may not reach damaging levels due to enhancement of antioxidant responses. However, inadequate exercise training (i.e., single bout of high-intensity or excessive exercise) may result in oxidative stress, muscle fatigue and muscle injury. Moreover, during the recovery period, impaired immunity has been reported, for example; excessive-inflammation and compensatory immunosuppression. Nutritional supplements, sometimes referred to as immuno-nutrients, may be required to reduce immunosuppression and excessive inflammation. Herein, we discuss the action and the possible targets of key immuno-nutrients such as L-glutamine, L-arginine, branched chain amino acids (BCAA) and whey protein.

**Electronic supplementary material:**

The online version of this article (doi:10.1186/s12970-014-0061-8) contains supplementary material, which is available to authorized users.

## Introduction

Elite athletes competing in national and international events are required to engage in multiple strenuous exercise training sessions to improve their performance. Although regular practice and moderate intensity exercise, for the general population, is essential to reduce the risk of chronic inflammatory diseases, athletes engaged in intense, prolonged or exhaustive physical exercise are more susceptible to the adverse effects from high-intensity exercise. Such effects include high rates of protein catabolism, a pro-inflammatory profile, accompanied by muscle damage, soreness, chronic oxidative stress [1] and immune suppression [2],[3]. A large number of studies have reported the harmful side effects (overtraining syndrome) and increased upper respiratory tract infection (URTI) promoted by exhaustive physical exercise [2],[4],[5].

**Figure 1:**
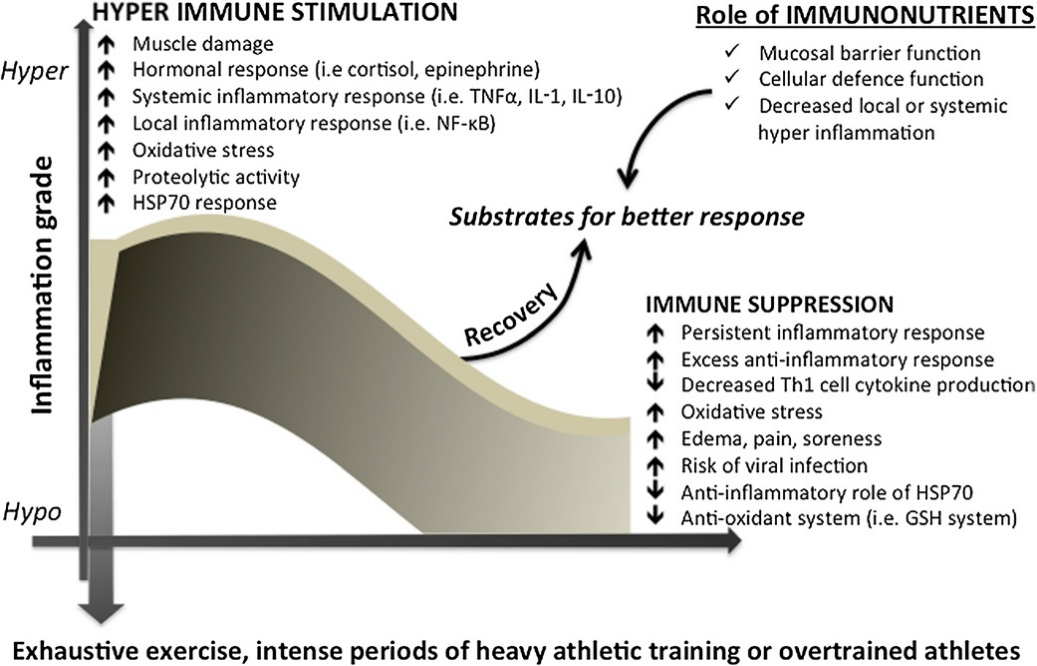
**Biphasic immuno-inflammatory response to severe exercise and the possible immunonutrition role.** Immuno-inflammatory response induced by severe exercise or heavy periods of training and the proposed role of specific nutrients with immune benefits, also called immunonutrition. Abbreviations: tumor necrosis factor-alpha (TNF-α), interleukin-1 (IL-1), interleukin-10 (IL-10), nuclear Factor-κB (NF-κB), glutathione (GSH), heat shock protein 70 kda (HSP70).

Although a balanced diet with high quality and sufficient quantity of nutrients is essential, there is growing evidence that some non-synthetic supplements can assist optimal nutrition. In fact, the use of nutritional supplements especially the provision of amino acids, has grown year-on-year [6]. There are few articles in the literature to address the topic of nutritional supplementation and immune consequences, from a metabolic and molecular standpoint. The use of proteins and amino acids for supplementation deserves special attention, since these molecules are critical for anti-oxidant and fuel provision, participating in the whole-body energy homeostasis, growth, development, recovery and immune responses. The key targets for immunonutrition may include provision of key metabolites for immune cells *per se*, the inflammatory response and cytokine release, the production of chaperone proteins such as the heat shock proteins (HSPs), redox balance (including glutathione, GSH metabolism), and protection of skeletal muscle mass (Figure 1).

### The evolution of immunonutrition

Key considerations that motivate athletes to consume nutritional supplements include: i) to improve their performance, ii) to strengthen immune function and, iii) to minimise the exercise recovery period [7]. The most widely used supplements are vitamins and minerals. Many studies have described the use of proteins, such as whey for supplements or isolated amino acids [8],[9]. Although the use of nutritional ergogenic aids in sports is topical, how and which nutrients may impact health and immune defense are interesting to the clinical nutrition field.

The role of nutritional support for immune function can be traced to 1810, when J. F. Menkel described that malnourished people in England presented with thymus atrophy. Other reports from the early 1900s, describe vitamin intervention studies [10] and reports exist from Ghetto physicians during World War II as to the poor health outcomes due to malnutrition [11]. More recently, positive outcomes related to total parenteral nutrition (TPN) administration, required during intensive medical care, have been described. These developments subsequently resulted in the formulation of products that could potentially modulate immune system activity, described as “immunonutrition” products. These interventions became popular for use with patients after 1990.

Most of the recent studies clearly demonstrate the importance of nutrients for trauma and surgical patients, as well as the frail elderly. Hence, strategies that include specific nutrients for enhanced immune function are frequently used in clinical nutrition therapy (e.g., for patients with burns, sepsis, cancer, HIV) and post-surgical situations using enteral or TPN routes. However, the concept of immunonutrition may be more widely applied, since the specific nutritional substrates for immune response can act on alternative targets, such as the gut mucosal barrier. Since athletes are at increased risk of upper respiratory tract infection (URTI), overtraining syndrome, chronic inflammatory response and oxidative stress [4], during and after periods of heavy exercise [12], immunonutritional approaches may be considered for future recommendations in the sport science field (Figure 1).

### Exercise-induced changes in the immune system: an overview

#### Changes in cytokine profile

Regular practice of moderate-intensity physical exercise has been shown to efficiently and positively impact physiological imbalances caused by different pathological situations. Exercise has been prescribed as a complementary therapeutic strategy in different modes of immunological dysfunction [13]. It has been clearly demonstrated that exercise induces considerable changes in immune function related to physiological responses to both metabolic and hormonal exercise-related alterations (Figure 1). Most of the exercise responses on the immune system are mediated by hormones such as adrenalin, cortisol, growth hormone (GH), and pro- and anti-inflammatory cytokines. The immunological changes are dependent on exercise intensity, type, and duration. For instance, cytokine production is modulated by a range of physiological stimuli that accompany exercise, such as stress hormones, energy crisis and oxidative stress [14]. In turn, exercise-induced cytokine effects depend on the type of mediator involved and the balance between pro-inflammatory cytokines (IL-1, TNF-α, IFNα, IFNγ, TNF-β, IL-2, IL-12, and MCP-1) and anti-inflammatory ones (IL-4, IL-10, IL-13, IL-12p40, IL-1ra).

During moderate intensity exercise, pro-inflammatory cytokine production is downregulated and anti-inflammatory cytokines, such as IL-1 receptor antagonist (IL-1ra), IL-10 and IL-6, are upregulated [15]-[17]. Strenuous and prolonged exercise induces increases in circulating TNF-α, IL-1β and IL-6 levels. This is counterbalanced by cytokine inhibitors (IL-1ra, sTNF-r1 and sTNF-r2) and the anti-inflammatory cytokine IL-10 [18]. The magnitude of the changes differs markedly depending on the cytokine being examined. For instance, plasma concentrations of IL-1 and TNF-α increase one-to two fold, whereas IL-6 has been reported to increase over 100-fold after prolonged exercise [18].

A large number of studies have reported increased plasma concentrations of anti-inflammatory cytokines, such as IL-1ra, IL-4 and IL-10, after various forms of exercise including brief maximal exercise [19], resistance exercise [19],[20], downhill running [21],[22], intense eccentric cycling [23], and endurance running and cycling [19],[24],[25]. Increased production of anti-inflammatory cytokines during exercise may serve to restrict pro-inflammatory reactions to exercise-induced muscle damage [23] and may also limit the production of pro-inflammatory cytokines associated with the development of ill states [26]. Conversely, increased production of anti-inflammatory cytokines during severe exercise may result in enhanced susceptibility to infections via alteration in the pro- vs. anti-inflammatory cytokine balance favoring an anti-inflammatory response [25].

Importantly, exercise induces robust increases in production and release of IL-6 [27],[28] from skeletal muscle. IL-6 then stimulates the appearance, in the circulation, of the anti-inflammatory cytokines IL-1ra and IL-10, and inhibits the production of the pro-inflammatory cytokine TNF-α [18],[26]. Hence, moderate exercise may decrease pro-inflammatory cytokine production while increasing anti-inflammatory cytokine production and action, which may induce a very strong anti-inflammatory cytokine response. The main modulator of these responses is likely the appearance of IL-6 in the circulation.

Another immune-regulatory protein that is now receiving considerable attention is HSP72. Studies have demonstrated HSP72 participation in conditions associated with inflammation such as type 1 (T1DM) and type 2 diabetes mellitus (T2DM), aging, and obesity [29]-[32]. HSP72 can induce different inflammatory responses according to its location (intra *vs.* extracellular) positioning this protein as a master regulator for the fine-tuned control of the immune system: while iHSP70 has anti-inflammatory effects, eHSP70 induce the opposite. Physical exercise is a very well known inductor of HSP70 expression [30],[33],[34]. Interestingly, some studies have demonstrated that exercise is a physiological stimulus that promotes an increase in the eHSP70 concentration [35],[36]. Both intensity and duration of exercise have effects as determined in plasma [37] and muscle samples [33],[34]. The rise in circulating levels of eHSP70 precedes any gene or protein expression changes in HSP70 in skeletal muscle [27],[34]. Additionally, acute exercise induces transient changes in the numbers and response of circulating lymphocytes which are considered a major eHSP70 source (nearly 100% of total eHSP70 release from the immune system) [38],[39].

#### Muscle damage, oxidative stress and inflammation

Activation of immune responses and adaptations after an acute exercise bout is related to muscle damage. Skeletal muscle damage that normally occurs after an acute and intense exercise bout is followed by a local inflammatory response that is “dose-dependent” on the intensity and duration of the exercise [40]. Moderate local inflammation is essential for the adaptation of the muscle, bone, and connective tissues [41]. The subsequent inflammation that occurs in response to the muscle damage is induced and intensified by the production of reactive oxygen and nitrogen species (ROS and RNS, respectively). Additionally, several cytokines (most pro-inflammatory), and molecules (histamine, serotonin and prostaglandins) are released, causing edema, pain and further inflammation until resolution and muscle recovery occurs [42]-[44]. Local inflammatory reactions may be induced during muscle cell apoptosis or necrosis by activated macrophages and by inflammatory cytokines [45].

The sources of ROS in exercise are many, for example, the activation of the superoxide generating NADPH-oxidase from immune cells that infiltrate the damaged area [46]. Elevated metabolism or enhanced mitochondrial activity (i.e. exercise), can continuously subject many tissue specific cells to insult from ROS and RNS. Intracellular O_2_^−^ may combine with NO to generate peroxynitrite, which may cause inhibition of activity of number of key signal transducing or metabolic enzymes [1]. Overproduction of ROS or a failure in intracellular defenses against ROS may stimulate molecular events resulting in disease [1]. There is a direct relation between muscle damage, neutrophil infiltration and ROS generation during the inflammatory process [43]. The free radical production during exercise has an essential role for signal transduction, the induction of cell damage, and for the initiation of the inflammatory response. Although the training results in a reduction of ROS through adaptations of the antioxidant systems, inadequate exercise training may result in changes in the redox status, oxidative stress [34],[44], muscle fatigue, and muscle injury [1],[47],[48]. In addition, during certain types of exercise (especially those involving eccentric contractions), there is a significant release of Fe^2+^ ions that may aggravate the oxidative stress due to chemical reactivity, culminating in muscle fatigue and damage [43].

Several muscle proteins, including actin, myosin, Ca^2+^ and K^+^ pumps are sensitive to the redox state, thus changes in ROS or RNS production can directly affect muscle contraction [49]. ROS and RNS can induce rises in intracellular Ca^2+^ (through interaction with Ca^2+^ channels) and also inactivation of several enzymes from anaerobic and aerobic metabolism, leading to muscle fatigue [50]. Since oxidative stress and excessive inflammation are related to the loss of muscle function, several strategies have been used to improve the muscle and immune cell redox status, using nutritional and anti-oxidant interventions [41].

#### Redox status: the target for immunonutrition?

Additionally to the previously cited redox-sensitive proteins, nuclear factor-κB (NF-κB) is extremely sensitive to the redox status of the cells [51]. This protein is a ubiquitous transcription factor originally discovered in B-lymphocytes, which is essential for inflammatory responses to a variety of signals, immune function, endothelial cell activation, and the control of cell growth. NF-κB is normally located in the cytoplasm in an inactive form bound to an inhibitory IκB protein. A wide variety of inflammatory stimuli (such as excessive ROS and RNS) can utilize specific signal transducing pathways to enable phosphorylation of IκB by IκB kinase (IKK) and thus ensure its proteasomal degradation [52]. IκB degradation will release NF-κB, allowing it to translocate to the nucleus and induce pro-inflammatory gene expression. In this way, our cells have very sensitive and responsive control mechanisms for regulating redox status and thus NF-κB activation, to regulate the optimal level of inflammation. The most important intracellular non-enzymatic antioxidants are GSH and its oxidative form GSSG (oxidized glutathione) [1],[53].

**Figure 2:**
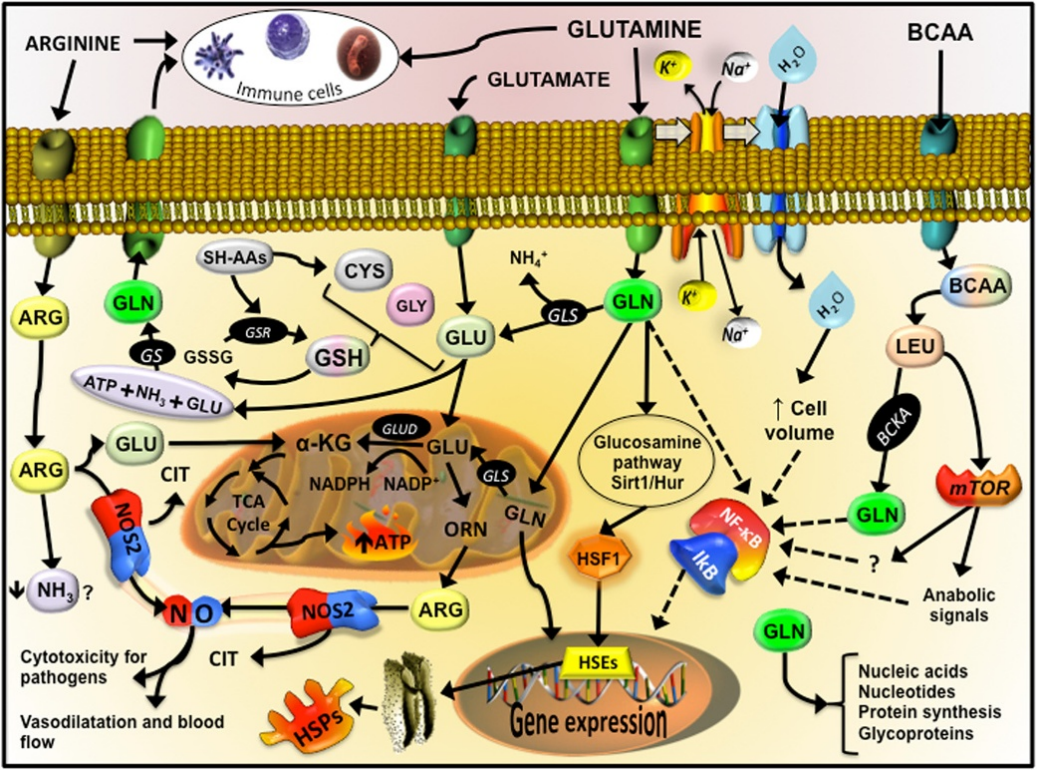
**Immune, antioxidant and inflammatory targets that L-glutamine, L-arginine and BCAA are involved.** From L-glutamine, glutamate (GLU) is produced through glutaminase activity (GLS), releasing ammonium ion (NH_4_^+^). Inside of mitochondria or in the cytosol, glutamate from L-glutamine, L-leucine (LEU) or L-arginine (ARG) is an important fuel (ATP) and/or precursor for the synthesis of intermediate metabolism of amino acids such as ornithine (ORN), antioxidant defenses such as glutathione (GSH), anabolic signals through mTOR cascade, and cell repair system such the as the heat shock proteins (HSPs). HSPs are modulated by the heat shock factor 1, which is activated by the glucosamine pathway, sirtuin 1 (Sirt1) and human antigen R (Hur), also known as nutrient sensors. *De novo* L-glutamine synthesis can occur through L-glutamine synthetase (GS), using glutamate, ATP and ammonia (NH_3_). L-glutamine is transported inside the cell trough active transport with sodium (Na^+^) potassium (K^+^) ATPase, which augment the absorption of water, altering the volume of the cell and stimulate the resistance to damage. L-arginine availability is important to NO production through nitric oxide synthase 2 (NOS2) and citrulline (CIT). Other Abbreviations: heat shock elements (HSEs); oxidized GSH (GSSG); GSH-S reductase (GSR); glutamate dehydrogenase (GLUD); alpha-ketoglutarato (α-KG).

GSH (γ-glutamyl-cysteinyl-glycine) is the predominant low-molecular-weight thiol (0.5-10 mmol/L) in animal cells. It is now well accepted that many forms of thiol oxidation (disulphide formation, gluathionylation and S-nitrosylation) are reversible and can provide a mechanism used by skeletal muscle cells in the regulation of metabolic signaling and transcriptional processes, including in muscle adaptation after exercise and training [1],[54]. Since the cellular redox state is crucial for several molecular pathways, and glutathione seems to be the key regulator/sensor for redox status, strategies aiming at improving GSH synthesis are now being studied. The synthesis of GSH from glutamate, cysteine, and glycine is catalyzed sequentially by two key cytosolic enzymes, γ-glutamylcysteine synthetase (GCS) and GSH synthetase (Figure 2). GCS is the key regulatory enzyme, activated by several types of stress including oxidative and nitrosative stress, inflammation, heat stress, and others [55]. It is therefore reasonable to speculate that amino acid and protein supplementation, may provide intracellular GSH precursors - an essential strategy to improve GSH synthesis and redox protection, leading also to better control of the inflammatory status and muscle recovery [56].

However, although antioxidant supplementation may at first be considered as beneficial, the consequent reduction of ROS/RNS could actually have negative effects in non-athletes. Muscle redox state may be best improved by providing skeletal muscle cells with the key natural precursors for GSH synthesis and allowing the cells to synthesize what they actually require. Exercise-induced ROS is not detrimental to human health, thus endogenous antioxidants may be sufficient to protect against exercise-induced oxidative damage, however this may not be applicable for elite athletes.

In addition to GSH metabolism, the levels of iHSP72 may also be involved in the control of exercise-induced muscle inflammation and adaptation [57]. Their expression has been shown to be induced by a wide range of stressors such as oxidative stress, thermal stress, hypoxia, viral infection, heavy metal contamination, ischemia, exercise metabolic stress and many others [33],[53]. As molecular chaperones, the HSP70 family can interact with other proteins (unfolded, in non-native state and/or stress-denatured conformations) to avoid inappropriately interactions, formation of protein aggregates and degradation of damaged proteins, helping the correct refolding of proteins. Other HSP functions include protein translocation, anti-apoptosis, and also anti-inflammatory response [58]. The anti-inflammatory role of the HSP70 is mediated by its interaction with the proteins involved in the activation of the NF-κB, blocking its translocation to the nucleus and slowing of the inflammatory process [51],[58]. Interestingly, specific amino acid supplementation has been shown to induce HSP70 and GSH in many cells, as will be described below.

### Immune mediating effects of L-glutamine

L-glutamine is probably the most widely recognized immuno-nutrient since it can be used as an oxidizable fuel, a substrate for nucleotide synthesis, a modulator of intermediary metabolism of amino acids [59],[60], HSP expression [33] and a component of GSH-mediated antioxidant defense (Figure 2) [44],[61], thus serving as a key substrate for cell survival, maintenance and proliferation. The use of L-glutamine as a nutritional supplement for sport and exercise increased in the 90′s, based on several clinical nutritional studies, that found benefits in attenuate the dramatic decrease in plasma and tissues L-glutamine levels [62], as well as immune cell function, including lymphocytes [8],[61] and neutrophils [59],[63]. Several important publications have described the importance of L-glutamine in clinical nutrition [59],[62],[64].

Oral L-glutamine supplementation (0.1 g/kg body wt) for athletes appeared to have a beneficial effect by attenuating the exercise-induced decrease in plasma L-glutamine levels [4],[65], the decreased number of lymphocytes, and eventually the risk of URTI’s [66]. Nevertheless, the efficacy of L-glutamine supplementation has raised many doubts and controversies, as subsequent studies with fixed (20–30 g/day) or variable (0.3 - 0.5 g/kg body wt) doses, or even in association with other macronutrients, such carbohydrates, did not report similar outcomes [12],[67],[68]. Possibly, for these reasons the last consensus statement in 2011 did not recommend L-glutamine supplements for sports and exercise [69].

The divergences between the clinical and sport nutrition data resulted on the idea that, perhaps, L-glutamine stores within the body cannot be sufficiently depleted by exercise [69]. Although, the evidences that L-glutamine is a direct modulator of the glutathione (antioxidant properties) and HSPs (with chaperone function and inflammatory control) synthesis (Figure 2) deserve some consideration. Furthermore, when L-glutamine is provided by oral or enteral ways in its free form, the amino acid is highly metabolized by the gut, fact that may explain the lower effect in other tissues and circulating cells, such as the immune cells. A possible alternative way is the exogenous administration of L-glutamine chemically attached to another amino acid (e.g. L-alanine), usually as a dipeptide, such L-alanyl-L-glutamine.

In humans [66] and animal models [70], acute oral L-glutamine supplementation, in its free form or as a dipeptide, is able to increase the plasma L-glutamine concentration between 30 to 120 minutes after ingestion. However, L-glutamine containing dipeptides are highly soluble and stable in solution, often used in enteral nutrition and TPN, and achieve high L-glutamine and L-alanine into the circulation. This effect has been attributed to the glycopeptide transport protein (PepT-1) in the intestinal cells (enterocytes), which have a more efficient transport mechanism for the absorption of dipeptides and tripeptides than for the absorption of free amino acids [71]. In this manner, L-glutamine from dipeptide administration can avoid metabolism by enterocytes, proceeding directly to the systemic circulation [47],[72], therefore increasing its availability to immune cells and other tissues [61]. In the dipeptide or in its free form, L-alanine can spare L-glutamine metabolism allowing the latter to be used by high-demand tissues [61].

*In vivo* studies have shown that L-glutamine supplements (free along with L-alanine and glutamine containing dipeptides) are able to increase the hepatic and muscular concentration of L-glutamine, which in turns increases the tissue concentration of GSH, attenuating the oxidative stress induced by long duration physical exercise [44]. This antioxidant effect is attributed to the supply of L-glutamate from L-glutamine, especially from plasma to immune cells and skeletal muscles [59],[60]. When transported inside the cell, L-glutamine simultaneously promotes the uptake of water, an increase in sodium ion Na^+^ uptake and the release of potassium ions (K^+^), which increase the cell hydration state and volume, which is important in the resistance to injury (Figure 2) [73]. L-glutamine availability increase neutrophil and lymphocyte activity and function [74], for example, generating NADPH for the NADPH oxidase enzyme [63], stimulating intermediary metabolism, and preventing apoptosis by maintaining mitochondrial function [8],[74],[75]. In fact, L-glutamine supplementation may attenuate muscle damage and inflammation (e.g. levels of TNF-α and PgE_2_) induced by exhausting exercise [47].

More recently, several studies have reported glutamine-enhanced stimulation of the HSP response induced by acute or chronic inflammation [34],[61]. L-glutamine activates intracellular nutrient sensors such as the sirtuins. Specifically sirtuin 1 (SIRT1)/human antigen R (HUR) may be activated through glucosamines [76] leading to activation of the heat shock transcription factor, HSF-1, and the heat shock elements (HSEs) in the nucleus [61], promoting cell survival [76]. SIRT1 acts on many substrates, including histones, forkhead box O (FOXO), NFκB and p53 [77]. Moreover, L-glutamine availability is a limiting step for mTOR complex 1 (mTORC1) activation pathway, a major regulator of cell size and tissue mass in both normal and diseased states [78]. Considering the highly evolutionarily conserved HSF-1-HSP70 response (known as the Stress Response), then the tight integration between metabolic (e.g., intermediary amino acid metabolism) and immune signaling leading to optimal responses against pathogens should not be unexpected. In summary, growing evidence in support of the immune mediating effects of L-glutamine, has resulted in an increase in interest for use in supplementation. More studies in athletes are required to determine optimal supplementation strategies, including the use of dipeptides with and without free amino acids.

### L-arginine- NO pathway

Nitric Oxide (NO) plays an important role in many functions in the body regulating vasodilatation and blood flow, inflammation and immune system activation, insulin secretion and sensitivity [79],[80], mitochondrial function and neurotransmission. The amino acid L-arginine is the main precursor of NO via nitric oxide synthase (NOS) activity, thus the availability of this amino acid may modulate NO production in conditions of competition for this amino acid (Figure 2) [81]. Dietary L-arginine and L-citrulline supplements may increase levels of NO metabolites. Although the effects of L-arginine supplementation has shown positive effects in many conditions such as diabetes [82] and cardiovascular diseases [83], this response has not been directly related to an improvement in performance related to sport and exercise [84]. Many of the positive aspects of L-arginine supplementation are related to improved circulation (due to increased NO levels) in sedentary individuals.

L-arginine supplementation in exercise training has not resulted in clearly defined outcomes. The high variability seems to be attributed to: i) human *vs.* animal models; ii) healthy *vs.* non-healthy subjects; iii) differences in body composition among subjects; iv) individual training status; v) duration of the supplementation and vi) type of exercise.

Although L-arginine can be produced by the adult human body (synthesized from L-glutamine, glutamate, and proline via the intestinal-renal axis in humans and most other mammals) [85], this amino acid is considered as a “conditionally essential” under conditions such as diabetes, additional ingestion may be required to normalize the plasma levels. L-arginine is a known powerful amino acid-based secretagogue for insulin, growth hormone (GH), glucagon and adrenaline [86]. Since this amino acid plays a critical role in cytoplasmic and nuclear protein synthesis, it has been used and suggested as an inductor of muscle growth and immune protection. L-arginine supplementation is known to increase the levels of both GH and IGF-1 in the blood but reduce IGFBP-3 protein levels [84]. However, most human studies have failed to show that L-arginine can provide improvements in performance in the sport and exercise context [87]-[90].

An increase in NO may result in improved blood flow and this could potentially be beneficial for individuals engaged in exercise training [90], by increasing nutrient delivery and/or waste-product removal from exercising skeletal muscles [90]. However, L-arginine, NO donors and NOS inhibitors induce effects on blood pressure, heart rate, and blood flow at rest conditions [83], several studies have shown that these agents have no effect on these variables during exercise in humans [83],[91]. Even though L-arginine supplementation increases blood flow in basal conditions, the amino acid does not change this variable during exercise. This could indicate that during exercise, other mechanisms of vasodilation in the microcirculation system of active muscles may be involved. There is evidence that vasodilatory prostanoids [92] may be important in determining responses to acetylcholine (Ach) in both diabetic [93] and non-diabetic subjects [94],[95], their effects mediated through an increase in cyclic AMP.

L-arginine supplementation may improve maximal (VO_2max_ test) exercise capacity in patients with cardiovascular disease [92],[96]. However, in healthy subjects, L-arginine-α-ketoglutarate did not influence body composition, muscular strength endurance, or aerobic capacity [97]. The finding that L-arginine-α-ketoglutarate supplementation did not improve aerobic capacity supports earlier studies that L-arginine improves VO_2max_ in various disease populations but not in healthy individuals [98]. In addition, L-arginine failed to improve muscular performance and recovery, independently of the training status [90].

Inadequate intake of dietary L-arginine may impair NO synthesis by both constitutive and inducible NOS in mammals [99], indicating a role for L-arginine in immune function. The effects of L-arginine supplementation on lymphocyte count has been reported [100], in a study which determined whether the transient hyperammonemia induced by high-intensity exercise (HI) could influence white blood cell distribution, and whether L-arginine could affect this parameter. Thirty-nine male jiu-jitsu practitioners were submitted to an acute bout of HI exercise using placebo or L-arginine (100 mg · kg-1 of body mass · day-1). Increases in lymphocyte number and ammonia were simultaneously reduced by L-arginine supplementation. Since the authors did not measure the pre-supplementation levels of L-arginine, it is difficult to know if the effect was induced by the higher levels of the amino acid or only by the correction of lower levels among the athletes.

In conclusion, it is clear that L-arginine supplementation improves exercise capacity and blood flow in conditions associated with endothelial dysfunction, such reduced basal NO production. However, in healthy individuals with normal levels of circulating NO, L-arginine supplementation has little or no effect.

### Multiple aspects of BCAA

From the nine amino acids nutritionally classified as essentials, three of these compounds are the branched chain amino acids (BCAA; L-valine, L-leucine and L-isoleucine). Mostly protein foods, such as meat, poultry, fish, eggs, milk and cheese can containing on average 15 to 20 grams of BCAA per 100 g of protein [101]. The presence of BCAA in the most primitive organisms that existed before the complex cellular evolution of higher organisms shows the importance this compounds to the metabolic evolution. BCAA are predominantly metabolized in the skeletal muscle, which means that they escape from liver metabolism and, after ingestion; they rapidly increase their concentration in plasma. Although the liver cannot directly metabolize BCAA, this tissue has an active system for the degradation of the α-branched-chain-keto acids (BCKA) derived from the corresponding BCAA [102] through the branched-chain α-keto acid dehydrogenase (BCKD), which contribute to gluconeogenesis [76].

Oxidative stress may be one of the underlying links between chronic inflammatory response and skeletal muscle wasting [102],[103], a fact that may negatively impact on macrophage and neutrophil function [74], as well as on lymphocyte proliferation [3]. Skeletal muscle cells have high activity of BCAA transaminases and L-glutamine synthetase, key enzymes in the synthesis of L-glutamine and other intermediary amino acids [12]. In this regard, when BCAA is present in the culture medium, lymphocyte proliferation capacity is increased; however, this most likely reflects an inability to synthesize sufficient amino acids and protein required for proliferation [104], which reinforces the important role of skeletal muscle in immune regulation. In animal [105] and human studies [106]-[108] under catabolic situations, such as infection or malnutrition, BCAA are crucial for the maintenance of immune function [104]. However, in catabolic but non-deficient situations, such as in elite athletes involved in heavy endurance or resistance training, the effects of BCAA administration is still not clear. When a large amount of protein is consumed, typically by athletes, an abundance of dietary BCAA will be available for metabolic and immune requirements (high-quality protein sources range from approximately 18-26% BCAA [109]).

In one study, acute and chronic BCAA supplementation (about 6 g/d) to *endurance* athletes resulted in attenuation of the fall in the plasma L-glutamine concentration and also modified the immune suppression promoted by the exercise [107]. Once stimulated through the supplementation of BCAA, cellular L-leucine uptake may enhance the synthesis and availability of L-glutamine by providing glutamate in the intracellular environment. Hence, it is believed that the immune effects of BCAA may be dependent on L-glutamine metabolism in the tissues, such as the skeletal muscle. In fact, in hyper-catabolic situations, such as burning, sepsis and malnutrition, BCAA administration can modulate inflammation through the L-glutamine pathway [110]. However, considering the effects of exercise, this pathway deserves some considerations. When lymphocytes are maintained *in vitro* in a low level of L-glutamine, identical to the lowest plasma L-glutamine concentration measured post-exercise (300 - 400 μM), these cells perform equally well [59] as when L-glutamine is added at a higher concentration similar to the resting plasma level (600 μM) [12]. Consequently, BCAA effects for sports and exercise with regard to immune function, may occur independently of L-glutamine synthesis and stimulation.

Some studies have reported that BCAA administration may attenuate higher inflammatory responses and muscle soreness induced by severe exercise. Prior to resistance squat exercise, BCAA supplementation (100 mg/kg body weight) was able to reduce the delayed-onset muscle soreness (DOMS) [111]. This effect is due to BCAA oxidation in tissues via generation of BCKA’s, such α-ketoisocaproate, α-keto-β-methylvalerate and α-ketoisovalerate derived from L-leucine, L-isoleucine and L-valine, respectively, and L-glutamine synthesis. BCAA supply and oxidation can inhibit the activity of pyruvate dehydrogenase, a key regulatory site between glycolysis and the citric acid cycle, a mechanism that favors the deviation of pyruvate to the formation of L-alanine which, after release, acts as a precursor in hepatic gluconeogenesis [112]. In fact, in animal studies, chronic supplementation with BCAA promoted a higher hepatic and muscle glycogen synthesis, even after an exhaustive exercise test [112]. L-leucine improved protein synthesis [105] through mTOR stimulation, hVpS34 and calcium-related proteins (Figure 2) [113], not during but after exercise activity [108]. This effect can limit the excessive activation of NF-κB, attenuating the uncontrolled inflammation and its effects, which include the DOMS.

Another possible protective mechanism of BCAA may be mediated through the antioxidant system. It has been shown that BCAA supplementation increased the expression of genes involved in the antioxidant defense, such superoxide dismutase (SOD) 1 and 2, catalase (CAT) and glutathione peroxidase 1 (GPx1) in trained middle-aged mice. Moreover, the same work reported reductions in oxidative stress in cardiac and skeletal muscle [110]. This led to the idea that redox balance can be a target for the potential benefits promoted by BCAA administration. In fact, BCAA and BCAA along with other sulphur-containing amino acids, such L-taurine, attenuated the DOMS and muscle damage induced by eccentric exercise [114].

The multiple aspects of BCAA, particularly L-leucine has shed light on their possible roles in metabolic disease. Of the BCAA only L-leucine has potent effects upon protein turnover (i.e. stimulates protein synthesis and inhibits protein degradation) via mTOR downstream pathways, thus inadequate ingestion of L-leucine may decrease relative concentrations of L-valine and L-isoleucine. This effect negatively impacts on protein turnover and is called L-leucine paradox, which may be explained by an imbalance of BCAA oxidation in the tricarboxylic acid cycle (TCA) via BCKD complex and anaplerosis reactions. The close relationship between BCAA and its participation in cell bioenergetics and oxidative metabolism may promote an insulinotropic effect in pancreatic β-cells [76]. Conversely, BCAA catabolism is associated with decreased insulin sensitivity in obese patients, fact that corroborates with animal models with excess intake of BCAA and lipids. In this scenario, BCAA catabolism, especially in muscle and liver would result in increased propionyl and succinyl CoA synthesis, leading to incomplete oxidation of fatty acids. In conclusion, while progress has been made, more studies are needed to establish the crosstalk between lipids and BCAA, as well as BCAA roles in metabolic dysfunctions [115].

### Whey proteins as an amino acid source

The constituents of milk have become recognized as functional foods, with direct impact on human health. Milk has two primary ‘fractions’ of proteins: caseins and whey. Whey is the liquid portion that represents ∼ 20% of the total protein content of bovine milk [116]. The advances in food processing, such ultrafiltration and microfiltration have resulted in the development of different whey protein products from dairy plants worldwide. The most well known whey proteins are: concentrate (about 80-95% of protein, with or without lactose), isolate (about 90-95% of protein, normally without carbohydrates), hydrolysed (smaller peptide fractions, reduce immunological reactions, such allergy) and non-denatured (native protein structures) [117]. Furthermore, whey proteins with casein, albumin and/or soy protein, commonly called blend products can be found in retail stores. For more details see Marshall [117] and Luhovyy, Akhavan [118].

**Figure 3:**
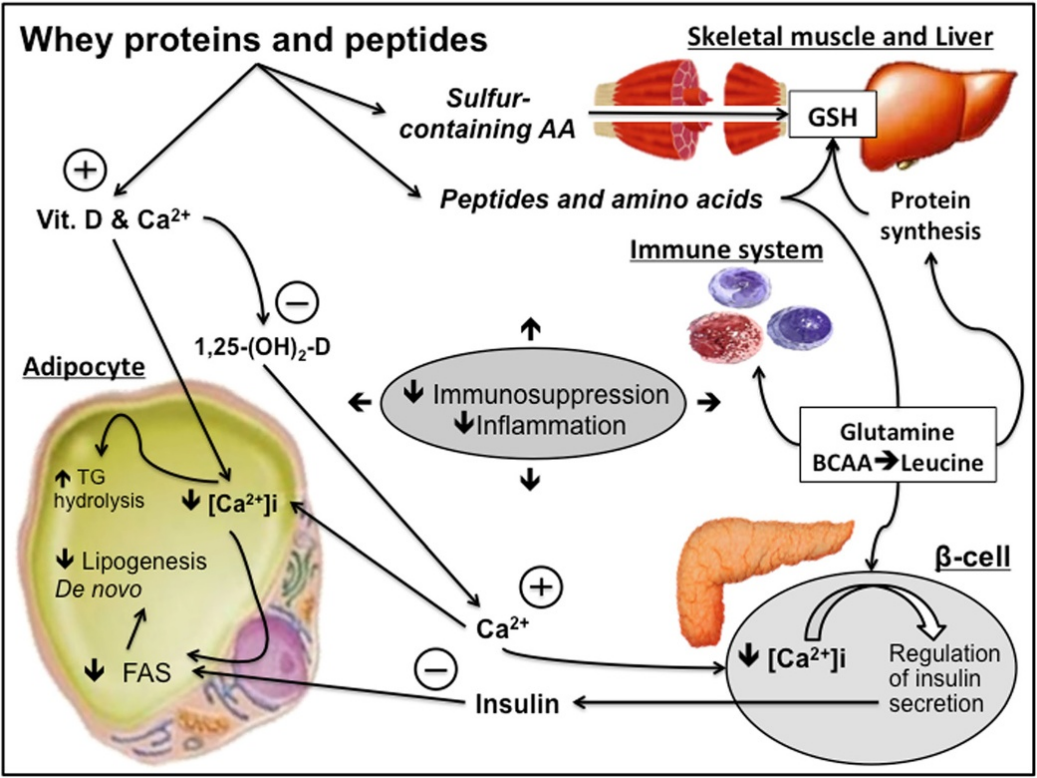
**Mechanisms involving whey proteins as a source of different immuno-nutrients.** Whey proteins can influence lipid metabolism, muscle protein synthesis/breakdown, antioxidant system, mediated by GSH. Abbreviations: Calcium (Ca^2+^), 1,25 Hydroxycholecalciferol (1,25-(OH)_2_-D), intracellular Calcium concentration ([Ca^2+^]i), Fatty Acid Synthase (FAS), Triacylglycerols (TGs).

Although whey proteins are considered as nutritional supplements, which means extra to the diet, the amino acid composition is very similar to that found in the skeletal muscles, providing almost all of the amino acids in approximate proportion to their ratios [119],[120]. Hence, these products are incorporated in the diet and not provided extra to the meal protein composition (e.g. meats plus whey). Accordingly, whey proteins it’s more likely a complement, than a supplement. Moreover, the components of whey include beta-lactoglobulin, alpha-lactalbumin, bovine serum albumin, lactoferrin, immunoglobulins (e.g. IgA), lactoperoxidase enzymes, glycomacropeptides, vitamins such as vitamin D, and minerals such as Ca^2+^[117],[121]. Lactoferrin and lactoferricin, demonstrate anti-microbial activity; lysosome, lactoperoxidase and diverse globulins and peptides provide a synergistic protective “cocktail” activity against viral and bacterial organisms [121]. In some chronic diseases with high inflammatory profile and adiposity, whey proteins have been used as adjuvant therapy acting in calcitropic hormones, such parathyroid hormone and 1,25 dihydroxycholecalciferol (1,25 - (OH 2)-D) [121]. Alone or combined with an exercise intervention whey studies demonstrate enhancements in energy loss through faecal fat excretion [122], regulation of glucose homeostasis [123] and adipogenesis [121], resulting in an anti- inflammatory effect (Figure 3) [124].

Whey protein supplements are considered also as a cocktail of amino acids, since they contain up to 26% of BCAA, plus L-arginine, L-lysine, L-glutamine, among others. Thus, the effects of whey protein in the immune system may represent the effect of particular amino acids *per se*. Moreover, whey proteins are rapidly digested and absorbed, resulting postprandial muscle protein synthesis [125],[126]. Several studies observed changes in muscle growth and performance increments with the chronic ingestion of whey protein supplements [121],[127],[128]. In one study, triathletes subjected to exhaustive exercise, exhibited a decreased mitochondrial transmembrane potential in both lymphocytes and neutrophils, which leads to apoptotic death and DNA fragmentation [8]. When whey protein enriched with L-glutamine is supplemented, this scenario is reversed, especially in lymphocytes [8], essential for the response against viral infections, such as URTI. On the other hand, the rapid absorption of whey products from the gut, and the hyperaminoacidemia is not the only critical characteristic for maximizing muscle protein synthesis. The time that amino acids are maintained in plasma is also important for the muscle protein turnover, providing gains in muscle mass. There are few studies comparing protein mixtures. Reidy, Walker [129] showed that a blend of whey and soy protein prolonged the elevation in blood amino acid levels after ingestion, when compared to whey protein alone, promoting a greater total muscle protein synthesis measured by the protein fractional synthetic rate (FSR). This is in agreement with other works, which found higher nitrogen retention, and less oxidation with whey blends combined with slowly digested protein, such as casein [130]. Stimulating post-exercise muscle protein synthesis and amino acid concentration maintenance, may also contribute to immune function however, more studies are needed.

The amino acid profile of whey protein supplements also includes sulphur-containing amino acids, such cysteine and taurine [121]. The high proportion of amino donors of sulfhydryl groups may attenuate the reduction of intracellular GSH concentration induced by intensive exercise [128]. Since immune cells, such as lymphocytes can be sensitive to a range of intracellular sulfhydryl compounds, such GSH and cysteine (Figure 2), whey supplementation may not only attenuate the oxidative stress induced by exercise but also help the maintenance of the redox status in immune cells. Experimental evidence support this mechanistic effect [117]. In a recent study, it was observed that the fall in the GHS content, in trained subjects submitted to an intense exercise program (4 weeks), have occurred in parallel with a decline in lymphocytes number. However, this scenario was reversed by N-acetyl-cysteine supplementation [131]. Furthermore, whey protein can act as an immune modulator through other mechanisms, such as L-glutamine, which is critical for the L-glutamine-GSH axis (Figure 3). Collectively, whey proteins via provision of an amino acid cocktail, exert per se an immune function through redox regulations pathways, and this seems particularly important in individuals engaged in intense and exhaustive exercise training programs, such elite athletes.

## Conclusion

Immunonutrition for clinical applications to sports activities represents an emerging area for health, especially regarding supply of proteins and amino acids, since they are required for the optimal synthesis and concentration of a variety of immune related proteins (including cytokines and antibodies). Amino acids will feed into and impact on the regulation of key metabolic pathways in immune cells and the cellular oxidative stress response. At the anti-inflammatory molecular level, new findings have been reported such as enhancement of HSP levels, NO synthesis, and GSH/GSSG regulation, all essential for optimal immune function and recovery from intense periods of training.

## Authors’ original submitted files for images

Below are the links to the authors’ original submitted files for images. Authors’ original file for figure 1 Authors’ original file for figure 2 Authors’ original file for figure 3
