# Supplementary figures and images for: Amino acid supplementation and impact on immune function in the context of exercise
Source: J Int Soc Sports Nutr. 2014 Dec 14;11:61. doi: 10.1186/s12970-014-0061-8 (PMC4272512; doi:10.1186/s12970-014-0061-8)

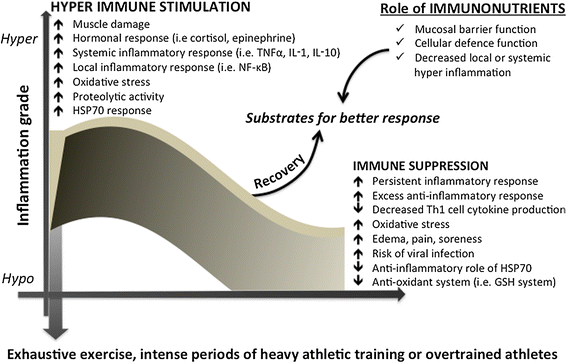

Supplement: Supplementary file 1 — Authors’ original file for figure 1 [file 12970_2014_61_MOESM1_ESM.gif]

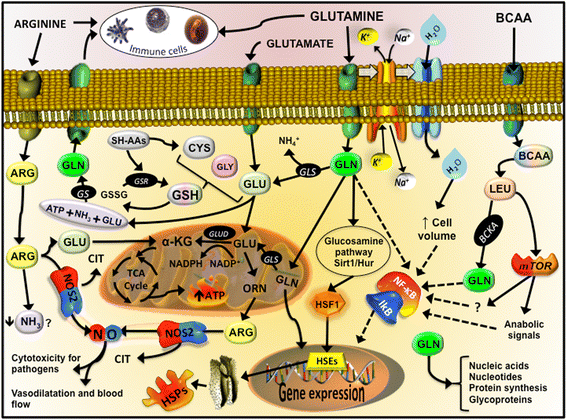

Supplement: Supplementary file 2 — Authors’ original file for figure 2 [file 12970_2014_61_MOESM2_ESM.gif]

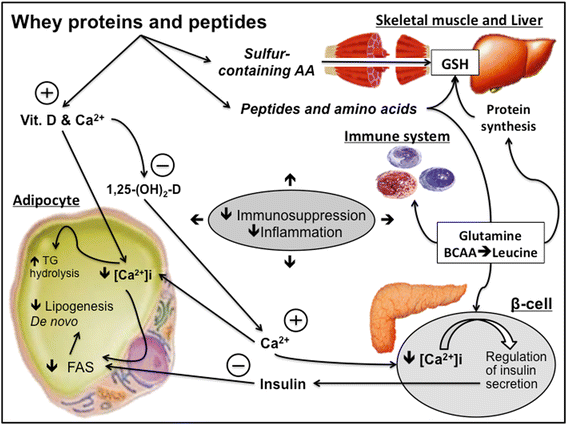

Supplement: Supplementary file 3 — Authors’ original file for figure 3 [file 12970_2014_61_MOESM3_ESM.gif]
